# A mean platelet volume in inflammatory bowel disease: A systematic review and meta-analysis

**DOI:** 10.1371/journal.pone.0273417

**Published:** 2022-08-30

**Authors:** Getachew Mesfin Bambo, Elias Shiferaw, Mulugeta Melku

**Affiliations:** 1 Department of Medical Laboratory Science, College of Health Sciences, Mizan Tepi University, Mizan, Ethiopia; 2 Department of Hematology and Immunohematology, School of Biomedical and Laboratory Sciences, University of Gondar, Gondar, Ethiopia; Babol University of Medical Science, ISLAMIC REPUBLIC OF IRAN

## Abstract

**Background:**

Inflammatory bowel disease (IBD) is a chronic gastrointestinal tract inflammatory state, which is affecting millions of individuals in the world. It can affect alimentary canals such as colon, rectum, ileum and other parts. In IBD, platelet parameters underwent several changes. Therefore, the aim of this review was determining the estimated pooled mean platelet volume and mean difference in inflammatory bowel disease to elucidate its potential diagnostic value.

**Methods:**

Articles were extensively searched in bibliographic databases using Medical Subject Heading and entry phrases or terms. In addition, articles were directly searched in Google Scholar to account for the studies omission in searching bibliographic databases. Observational (cohort, cross-sectional and case-control) studies, published in English language and conducted on IBD were included. For studies meeting the eligibility criteria, the first author’s name, publication year, population, study design, study area, sample size, mean platelet volume and standard deviation were extracted and entered in to Microsoft-excel. The analysis was done by Stata version 11. In order to estimate the pooled mean platelet volume and mean difference, random effect model was done. The heterogeneity was quantified using Higgin’s I^2^ statistics. Publication bias was determined using Egger’s test statistics and funnel plot. Sub-group analysis based on population carried to reduce heterogeneity.

**Results:**

A total of 17 relevant articles with 2957 participants (1823 IBD cases and 1134 healthy controls) were included to this study. The pooled estimated MPV was 9.29fl; 95% CI: 9.01–9.57 and 9.50fl; 95% CI: 8.81–10.20 in IBD and control groups, respectively. The standardized pooled estimate of mean difference in mean platelet volume was -0.83fl; 95% CI: -1.15, -0.51; I^2^: 93.1%; P-value < 0.001. In subgroup analysis based on population, the highest estimated mean difference in MPV was observed among patients of CD; -2.30; 95% CI: -3.46, -1.14; I^2^: 97.8%; P-value < 0.001.

**Conclusion:**

According to the current systematic review and meta-analysis, mean platelet volume was lower in IBD compared to control. The decreased mean platelet volume could be attributed to platelet consumption or sequestration associated with the progression of IBD. As a result, in IBD, mean platelet volume can provide diagnostic and prognostic information.

## Introduction

Inflammatory bowel disease (IBD) is a complex disorder which instigated and amplified by the co-influence of genetic and environmental variables that perturb the immune micro-biome axis against luminal bacteria [1]. In developing country, parasites such as Blastocystis species and Giardia lamblia, are the leading causes for IBD [2]. It is mainly idiopathic disorder which can be caused by excessive and chronic inflammation of gastro-intestinal tract (GIT) that leading for rectal bleeding and weight loss [3]. Environmental and genetic factors such as altered luminal bacteria and increased intestinal permeability lead for a dysregulated immune system, resulting to gastrointestinal damage. It includes ulcerative colitis (UC) and Crohn’s disease (CD), but the chronic inflammation may not only be due to the immune system. The CD can affect all parts of alimentary canal and UC, primarily colon and the rectum. Appendical CD is rare and indistinguishable from acute appendicitis (AA) [4].

Severity of IBD depends on segment of the intestine involved. A complication or different disease presentations were intra and extra intestinal complications, such as bowel perforation, massive hemorrhage, abdominal abscess, fistula, malignancy, rectal bleeding, abdominal pain, constipation and hepatobiliary disease. Fistulas are typical complication of CD [5, 6]. Inflammatory bowel disease affects millions of individuals in the world. In approximation, about 25% of IBD patients are common before the age of 18 [7]. It is associated with comorbidity with novel virus, covid-19.

Indeed, gut-related micro vascular dysfunction in IBD leads to decreased vasodilation capacity and tissue hypo-perfusion as well as lower rate of mucosal healing and refractory inflammatory ulcerations related with Covid-19 infection [8]. Furthermore, chronic inflammation increases the risk of venous and arterial thrombosis in IBD patients infected with the severe acute respiratory syndrome coronavirus 2 (SARS-CoV-2) [9]. It is now clear that comorbidities are associated with poorer clinical outcome in IBD patients with Covid-19 [10, 11]. Prevalence of comorbidities in this group of patients: about 60% have two or more comorbidities, which has an effect on treatment selection and disease outcomes [12].

Platelets contribute to the inflammatory process, microbial host defense; wound healing, angiogenesis and remodeling in addition to their essential role in hemostasis and thrombosis (7). Mean platelet volume (MPV),plateletcrit (PCT) and PDW are a set of platelet parameters collectively calculated by the automated complete blood count (CBC) profile [13, 14]. Platelets play a role in inflammation in a number of diseases, according to a wide body of evidence. In addition, recent research has found a correlation between platelet indices and inflammation. In IBD, there were several changes in platelet parameters, increased scale activity, density, platelet distribution width (PDW), PCT, and granulation augmentation. Moreover, the platelet releases a significant amount of pro-inflammatory substances when triggered at inflammatory sites [15–18]. Furthermore, most research showed thrombocytosis and lower MPV in CD patients (9.9fl) relative to healthy population (10.9fl) [19]. There is also evidence of reduced MPV in recurrence (7.8fl) and remission of CD (8.33fl) [20].

In current clinical practice, non–invasive biomarkers such as C-reactive protein (CRP) erythrocyte sedimentation rate (ESR) are commonly used as important for both early diagnosis and accurate monitoring of the disease activity in IBD patients [21–24]. As an acute phase protein, CRP is widely available and inexpensive diagnostic test; but, elevated serum CRP levels can also be affected by other extra intestinal inflammatory processes, and also there is genetic variability in the CRP production of individuals [25]. Moreover, because of the delayed reaction to disease conditions, ESR testing is less commonly used than CRP [21, 23]. Fecal calprotectin also has comparatively increased sensitivity and specificity and is the ideal clinical and biological marker for IBD evaluation [24, 26].

Tissue biopsy commonly used to confirm an IBD diagnosis for inflammation and changes in tissue architecture. However, CD is a transmural, a biopsy limited to the sub-mucosa that does not necessarily reflect disease activity in the deeper layers of the bowel wall. This type of test is invasive and it is less likely to be required if there is no inflammation [27]. It is fact that MPV has been used as a diagnostic marker in CRC [26, 28]. The diagnosis of an inflammatory disease is controversial and non-specific. In addition, there is wide disagreement over high costs, long-term requirements and low efficacy. In contradiction to this, the MPV test is low cost and more available in CBC.

It is fact that there is no gold standard diagnostic test to examine the severity and disease activities of IBD. In addition, there is wide disagreement over high costs, long-term requirements and low efficacy. However, MPV test is low cost and more available in CBC. Furthermore, there is no established guideline for considering MPV as diagnostic test in IBD. Therefore, cumulative evidence would be important to elucidate the diagnostic and prognostic values of MPV in IBD. In this regard, the main objectives this systematic review and meta-analysis were determining the pooled MPV and mean difference in IBD compared to health control to elucidate its potential diagnostic value. Hence, this systematic review and meta-analysis will provide sufficient evidence based information of MPV in IBD.

## Methods

### Design and protocol registration

This systematic review and meta-analysis was designed in accordance with Preferred Reporting Items for Systematic Review and meta-analysis Protocols (PRISMA-P) 2015 guide lines [29]. Pocket studies conducted on IBD were used to conduct this study to determine the pooled MPV and mean difference considering the CoCoPop (condition, context, population). The protocol was registered in the Prospero database under registration number CRD42021238610.

### Inclusion criteria and exclusion criteria

#### Inclusion criteria were

1) observational community and institutional based studies conducted on CD, AA, UC, general IBD and protocolits and 2) studies that reported MPV with standard deviation (SD) or confidence interval (CI).

#### Exclusion criteria were

1) case reports and abstract without full-length articles and 2) articles conducted on CRC, gangrenous appendicitis, drug efficacy, omitted generalizability.

#### Sources of data

Relevant articles were found using major electronic databases (PubMed, MEDLINE, HINARI, Embase, Scopus, Cinahl, African online archives and other source like Google scholar. In addition to account for the studies omission during electronic database searches, a direct Google search was carried using listed reference in included articles.

#### Searching strategy

We identified the entry terms by using MeSH (Medical subject heading) browser. Appropriate MeSH phrases and searching terms were merged using the Boolean operators "OR" and "AND" to fit the advanced searching. We used (“mean platelet volume” [Title/Abstract] OR “platelet, mean volume” [Title/Abstract]) OR “platelets, mean volume” [Title/Abstract] OR “volume, mean platelet” [Title/Abstract]) OR “volumes, mean platelet” [Title/Abstract] OR ‘‘MPV”[Title/Abstract] AND “platelet parameters” [Title/Abstract] AND “inflammatory marker” [Title/Abstract] AND “bowel disease” [Title/Abstract] AND “ulcerative colitis” [Title/Abstract] AND “Crohn’s disease” [Title/Abstract] AND “appendicitis” [Title/Abstract] for searching articles in PubMed. The above-mentioned entry terms were also extensively searched separately.

#### Outcome of interest

Primary outcome of interest was the pooled SMD in MPV in IBD versus control. Second, we determined the pooled MPV in IBD and healthy controls.

#### Study selection

Three independent authors (MM, ES and GM) identified the articles from reputable data bases and other sources. Searched articles were combined to Endnote X7 and duplicates were removed. Using inclusion criteria, two reviewers (GM and ES) evaluated the title, abstract and then full-text review for data abstraction. Any disagreements between two independent reviewers (GM and ES) were settled by (MM) in order to reach a consensus.

#### Quality assessment of the included studies

Three independent reviewers (MM, ES and GM) investigated the quality of the articles. The full texts of the articles were used to evaluate whether the study met the selection criteria or the article’s eligibility was in doubt. Methodological validity was assessed for each study design using the quality evaluation tool of Joanna Brigg Institute (JBI) criteria [30]. The JBI check list of related items, sampling, eligibility protocols, description of study subject and setting, appropriate statistical analysis, case definition, confounder identification, valid and reliable result measurement, bias minimization, comparability in study participants and generalizability the of study were checked. The scoring system were 0 (not done), 1 (done), UC (unclear), NA (not applicable) and the judgments of score range for cross-sectional, 0 (lowest quality) to 8 (highest quality), for case-control, 0 (lowest quality) to 10 (highest quality) and for cohort 0 (lowest quality) to 11 (highest quality) [30]. Articles, scored average of ≥50% were included in this meta- analysis (S1 Table).

#### Data extraction

After the assessment of the methodological and the allover characteristics of studies, data items were subjected to data extraction via data extraction Microsoft Excel sheet. For each articles, meeting the eligibility protocol, first author’s name, total sample size, publication year, population, study design, study area and results such as, average value of MPV and SD were extracted for each article.

### Data synthesis

Random and fixed effect models were conducted using Stata version 11 to estimate the pooled MPV and mean difference in MPV in IBD patients and control. Due to substantial heterogeneity, random effect model was used. Standardized mean difference (SMD) as measurement scale was used to determine the difference in MPV at 95% CI. The degree of heterogeneity with in each study was tested by using Higgins’s I^2^ statistics showing the magnitude of heterogeneity [31]. Statistically significant heterogeneity was found to be I^2^ > 50%. Sensitivity analysis was conducted to identify disproportionately influencing the results. Subgroup analysis by population character was employed to resolve substantial heterogeneity. Publication bias and small study effects were estimated using funnel plot and Egger weighted test. A p-value < 0.05 was considered for evidence of significance in publication bias [32].

## Results

### The review process and description of included studies

**Fig 1:**
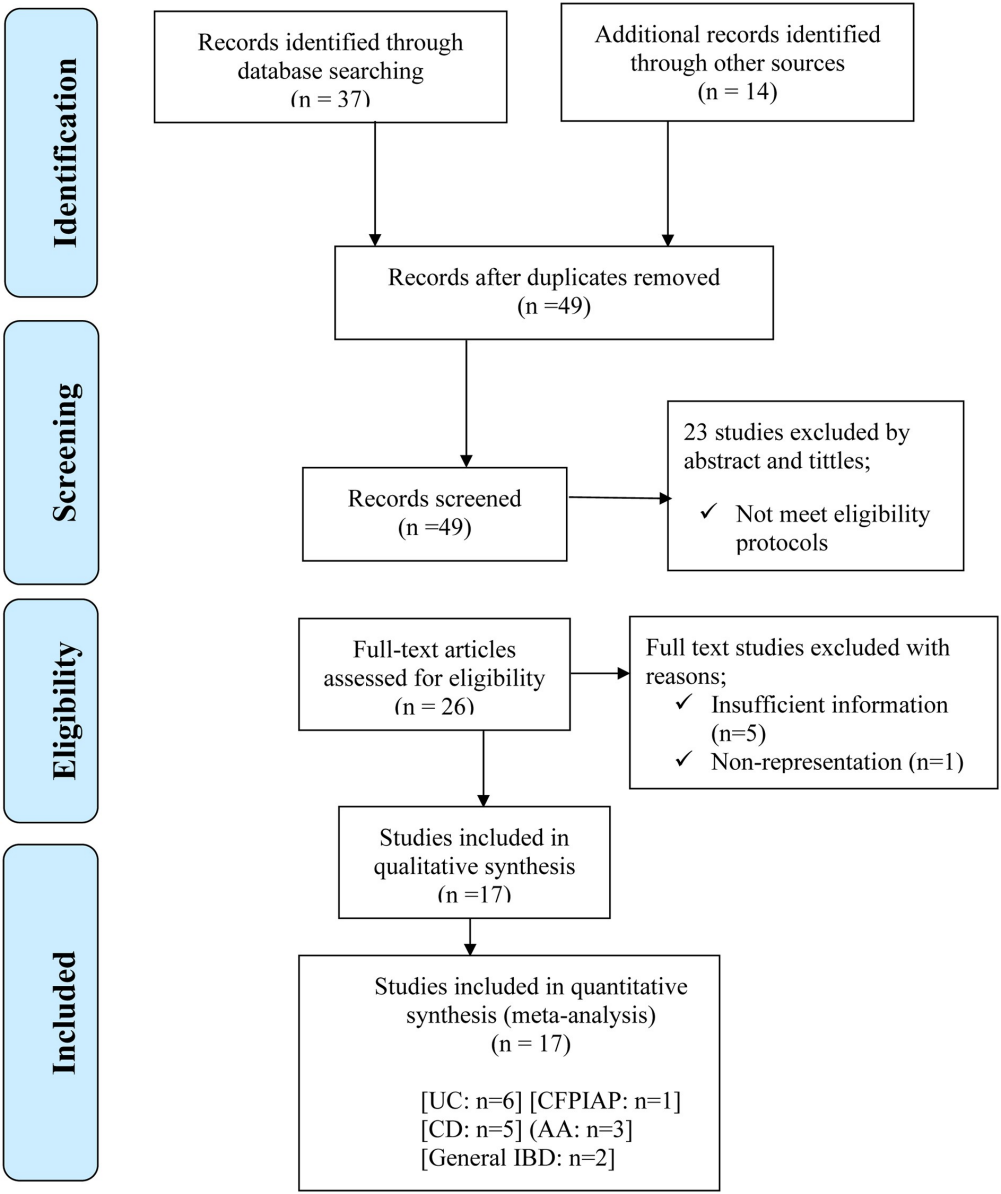
PRISMA flow chart describing screening protocols of studies for Meta-analysis. NB; UC: Ulcerative Colitis; CD: Crohn’s Disease; IBD: Inflammatory Bowel Disease; AA: Acute Appendicitis; CFPIAP: Child Food Protein Induced Allergic Proctocolitis.

Total of 51 studies, 5 abstracts and 46 full-text original articles were retrieved after searching the databases and other sources. After two duplicate studies removed [33, 34], 18 articles were discarded owing to irrelevant tittles. Eight articles were discarded after reading their abstracts. Twenty three articles were eligible for full-text review. Finally, 17 articles met the inclusion criteria (Fig 1). A total of 2,957 participants were involved from 17 included articles (1823 IBD patients and 1134 healthy controls). Six of them contained information on the; MPV for evaluation of disease activity in IBD, platelet indices in UC, coagulopathy in IBD, formation of platelet aggregation in IBD and neutrophil to lymphocyte ration in IBD (5 prospective cohort studies and 1comparative cross-sectional study) [35–40]. Five articles were, for the evaluation and investigation of platelet indices as a useful marker on CD (4 cohort and 1 retrospective cross-sectional) [36, 38, 39, 41, 42] (Table 1). Of those included articles, 2 were conducted for the investigation of MPV as useful biomarkers in general IBD (1 cross-sectional and 1 cohort) [35, 43] (Table 1).

**Table 1:** Summary characteristics of studies included in meta-analysis.

S.N	Author (reference)	Year	Population	Study area	Study design	MPV		Sample size		SD	
						IBD	HC	IBD	HC	IBD	HC
1.	Irving P et’ al [35]	2004	IBD patients	UK	Cross-sectional	8.4	8.95	67	20	1.05	0.78
2.	Öztürk Z et’al [36]	2013	CD patients	Turkey	Prospective cohort	8.23	8.98	72	40	1.32	1.34
3.	Öztürk Z et’al [36]	2013	UC patients	Turkey	Prospective cohort	8.38	8.98	103	40	1.24	0.98
4.	Kayahan H et’al [37]	2007	Active UC patients	Turkey	Prospective cohort	7.91	9.18	16	20	0.9	1.02
5.	Kayahan H et’al [37]	2007	Inactive UC patients	Turkey	Prospective cohort	8.77	9.18	21	20	0.92	1.02
6.	Kapsoritakis A et’al [38]	2001	UC patients	Greek	Prospective cohort	8.5	9.4	93	38	0.9	1.20
7.	Kapsoritakis A et’al [38]	2001	CD patients	Greek	Prospective cohort	7.8	9.4	66	38	1.00	1.20
8.	Yuksel et’al [39]	2009	UC patients	Turkey	Comparative cross-sectional	8.29	8.65	61	27	1.02	0.79
9.	Shen J et’ al [40]	2009	UC patients	China	Prospective cohort	9.83	10.74	195	116	1.9	1.03
10.	Shen J et’ al [40]	2009	CD patients	China	Prospective cohort	9.52	10.74	76	116	1.93	1.03
11.	Liu S et’al [41]	2012	CD patients	China	Retrospective cross-sectional	9.55	11.1	61	50	0.2	0.16
12.	Tang J et’ al [42]	2015	CD patients	China	Retrospective cohort	9.92	10.44	130	130	0.9	0.89
13.	Dogan Y et’al [43]	2011	IBD patients	Turkey	Prospective cohort	8.51	8.58	69	38	1.34	1.34
14.	Nacaroğlu H et’ al [44]	2018	Allergic proctocolitis patients	Turkey	Retrospective cross sectional	8.89	8.12	46	13	0.9	0.96
15.	Tanrikulu C et’ al [45]	2014	AA patients	Turkey	Retrospective cross-sectional	7.75	8.49	260	158	1.2	0.97
16.	Ceylan B et’ all [46]	2016	AA patients	Europe	Retrospective case-control	9.78	10.2	192	170	0.9	1.21
17.	Dinc B et’al [47]	2015	AA patients	World journal	Retrospective, case-control	8.5	8.9	295	100	0.7	0.92

**NB**: AA: Acute appendicitis; **CD**: Crohn’s Disease; **UC**: Ulcerative Colitis, **IBD**: Inflammatory Bowel Disease; **MPV**: Mean Platelet Volume; **HC**: Healthy control, **SD**: Standard Deviation and **UK**: United Kingdom. **Year**: indicates year of publication

One was conducted to establish the relationship between neutrophil-to-lymphocyte ratio and MPV with the diagnosis and development of child food protein-induced allergic proctocolitis (FPIAP) tolerance in infants (retrospective cross-sectional) [44]. Three were conducted for new diagnostic marker parameters for MPV, PDW in appendicitis (2 case-controls and 1 cross-sectional) [45–47]. Of the research included, nine articles (52.9%) were published after 2010 [36, 41–47], whereas eight (47.06%) before 2010 [35, 37–40] (Table 1).

### Publication bias

**Fig 2:**
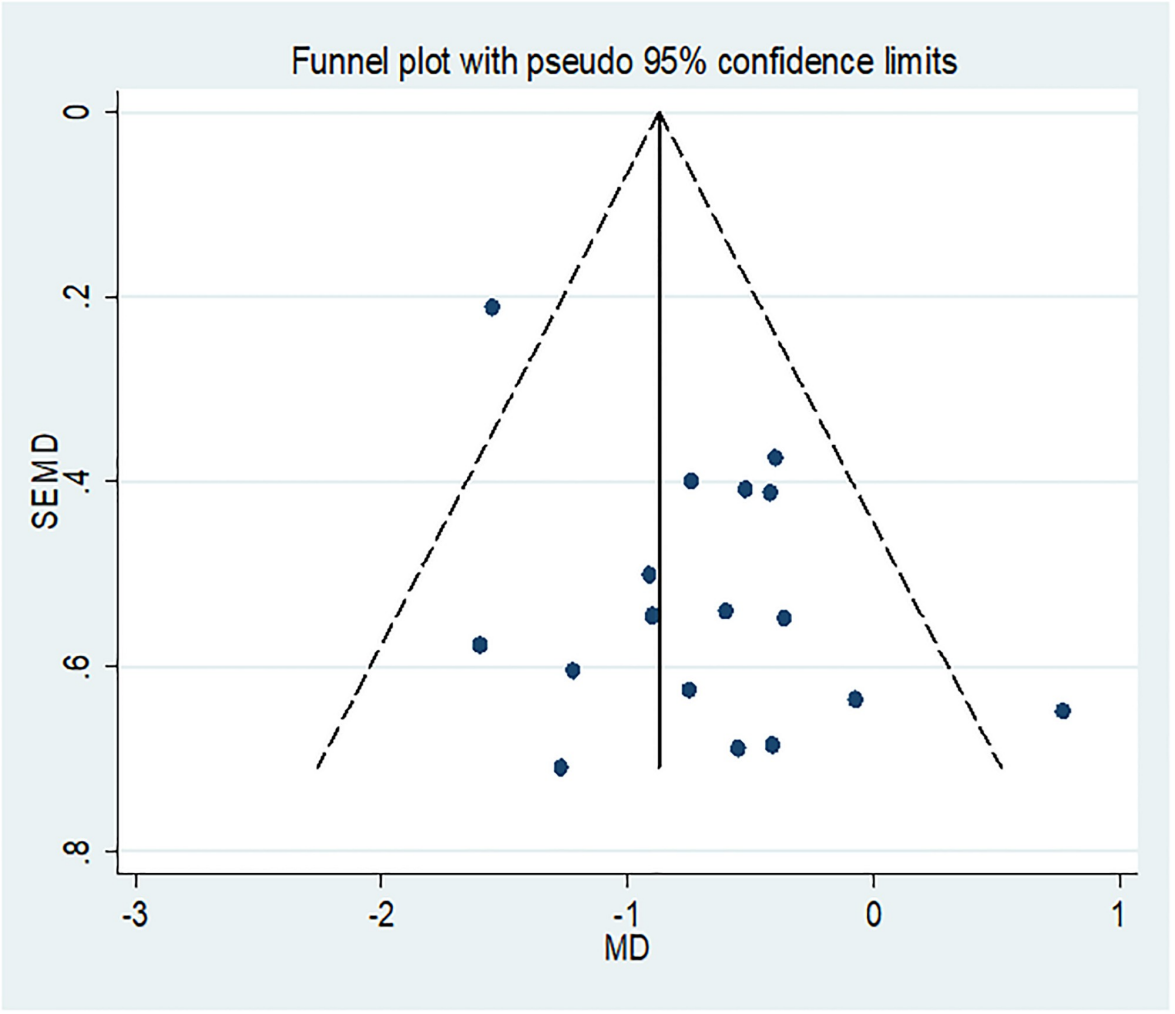
Funnel plot showing publication bias of included articles. Dot on the black line represents each individual article. Y-axis shows the standard error of mean difference (SEMD). The x-axis shows estimate mean difference (MD) of the included articles.

Potential publication bias was assessed by using funnel plot and egger’s statistics. The Egger’s test for publication bias was marginally insignificant (p = 0.59), suggesting that there was no indication of publication bias in included articles (Table 2). Furthermore, a funnel plot was used to demonstrate the existence or absence of publishing bias. Included studies seem symmetric and felled within the triangular region of funnel (Fig 2).

**Table 2:** Egger’s test statistics.

	*Coef*.	*Std. Err*	*t*	*P>|t|*	*[95% Conf. Interval]*
**Slope**	4.44	0.21	21.28	0.000	2.90–7.53
**Bias**	0.01	0.13	0.55	0.59	-0.04–0.06

**Std Eff**: Standard Effect; **Coef: Coefficient**:**T**: T- test Statistics; **Std. Err**: Standard Error; **P**: P-value of significance by assuming null zero value; **Conf. Interval**: Confidence Interval.

#### Quality and heterogeneity test

Regarding the quality, most of the studies scored high quality, greater than 75%. For individual studies, quality was assessed by using JBI critical appraising tool to minimize risk of bias. Each item was assessed for grading the articles as poor quality (<50%) good (50–75%) and high quality (>75%) (S1 Table). Included studies exhibited the substantial heterogeneity (I^2^, 93.1%; p < 0.001) in random model effect analysis of mean difference of MPV (Fig 5). To reduce the substantial heterogeneity, subgroup analysis, in difference in MPV based on population was done. The result showed no substantial heterogeneity in studies conducted on UC, general IBD, AA (I^2^; 33.7%, 33.9% and 37.9%; p = 0.18, 0.22 and 0.20) respectively, but still substantial heterogeneity was observed in studies conducted among CD (I^2^; 97.8%; p < 0.001) (Fig 6).

#### Sensitivity analysis

Individual study had a negligible impact on the pooled estimate, indicating the robustness of the aggregated estimate. When examining the pooled MPV differences by ignoring one study at a time, the results were consistent and accurate (Table 3).

**Table 3:** Sensitivity analysis of SMD in MPV.

S/no	Study omitted	Estimate	95%[Conf. Interval]	I^2^	P-vale
1.	Irving P et’ al (2004) [35]	-0.85	-1.19–0.52	93.6%	< 0.001
2.	Öztürk Z et’al (2013) [36]	-0.85	-1.20–0.52	93.8%	< 0.001
3.	Öztürk Z et’al (2013) [36]	-0.86	-1.195–0.52	93.4%	< 0.001
4.	Kayahan H et’al (2007) [37]	-0.80	-1.14–0.48	92.8%	< 0.001
5.	Kayahan H et’al (2007) [37]	-0.85	-1.20–0.52	92.5%	< 0.001
6.	Kapsoritakis A et’al (2001) [38]	-0.78	-1.11–0.46	93.4%	< 0.001
7.	Kapsoritakis A et’al (2001) [38]	-0.83	-1.14–0.49	93.9%	< 0.001
8.	Yuksel et’al (2009) [39]	-0.86	-1.20–0.53	92.6%	< 0.001
9.	Shen J et’ al (2009) [40]	-0.86	-1.21–0.51	92.5%	< 0.001
10.	Shen J et’ al (2009) [40]	-0.84	-1.18–0.50	93.5%	< 0.001
11.	Liu S et’al (2012) [41]	-0.56	-0.73–0.39	93.6%	< 0.001
12.	Tang J et’ al (2015) [42]	-0.86	-1.21–0.51	92.4%	< 0.001
13.	DoganY et’al (2011) [43]	-0.88	-1.20–0.55	92.9%	< 0.001
14.	Nacaroglu H et’ al (2018) [44]	-0.91	-1.24–0.60	93.8%	< 0.001
15.	Tanrikulu C et’ al (2014) [45]	-0.86	-1.23–0.51	92.8%	< 0.001
16.	Ceylan B et’ all (2016) [46]	-0.88	-1.23–0.53	92.8%	< 0.001
17.	Dinc B et’al (2015) [47]	-0.86	-1.22–0.52	92.6%	< 0.001
**Combined**		-0.83	-1.15–0.51	93.1%	< 0.001

**I**^**2**^: I-squared; illustrates the heterogeneity between studies; **Conf.Interval**: Confidence Interval; P-value indicates statistical significance of heterogeneity.

### Pooled estimated MPV and mean difference in MPV

#### The pooled estimated MPV in IBD and healthy control groups

**Fig 3:**
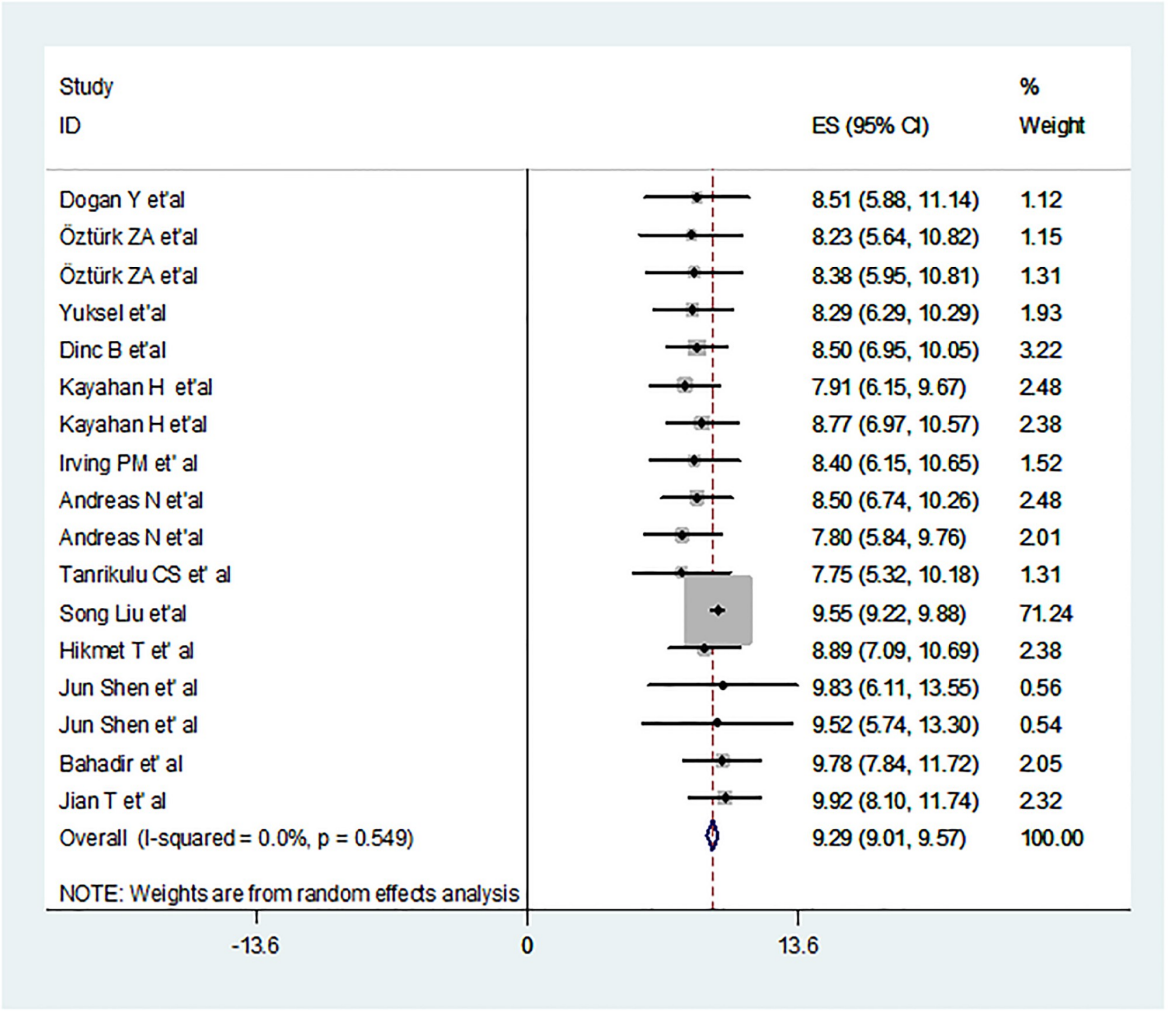
Forest plot of pooled estimated MPV in IBD patients using random effect model. The size of the x-axis shows the estimate pooled MPV of the studies. In the pooled point calculation, the dotted line represents the MPV. The black dot in the middle of the gray box reflects the estimate pooled MPV of each studies point and the line shows the 95% CI of the estimates. The gray boxes represent each study weight that contributes to the estimation of the pooled MPV. I-squared illustrates the heterogeneity between the included studies.

**Fig 4:**
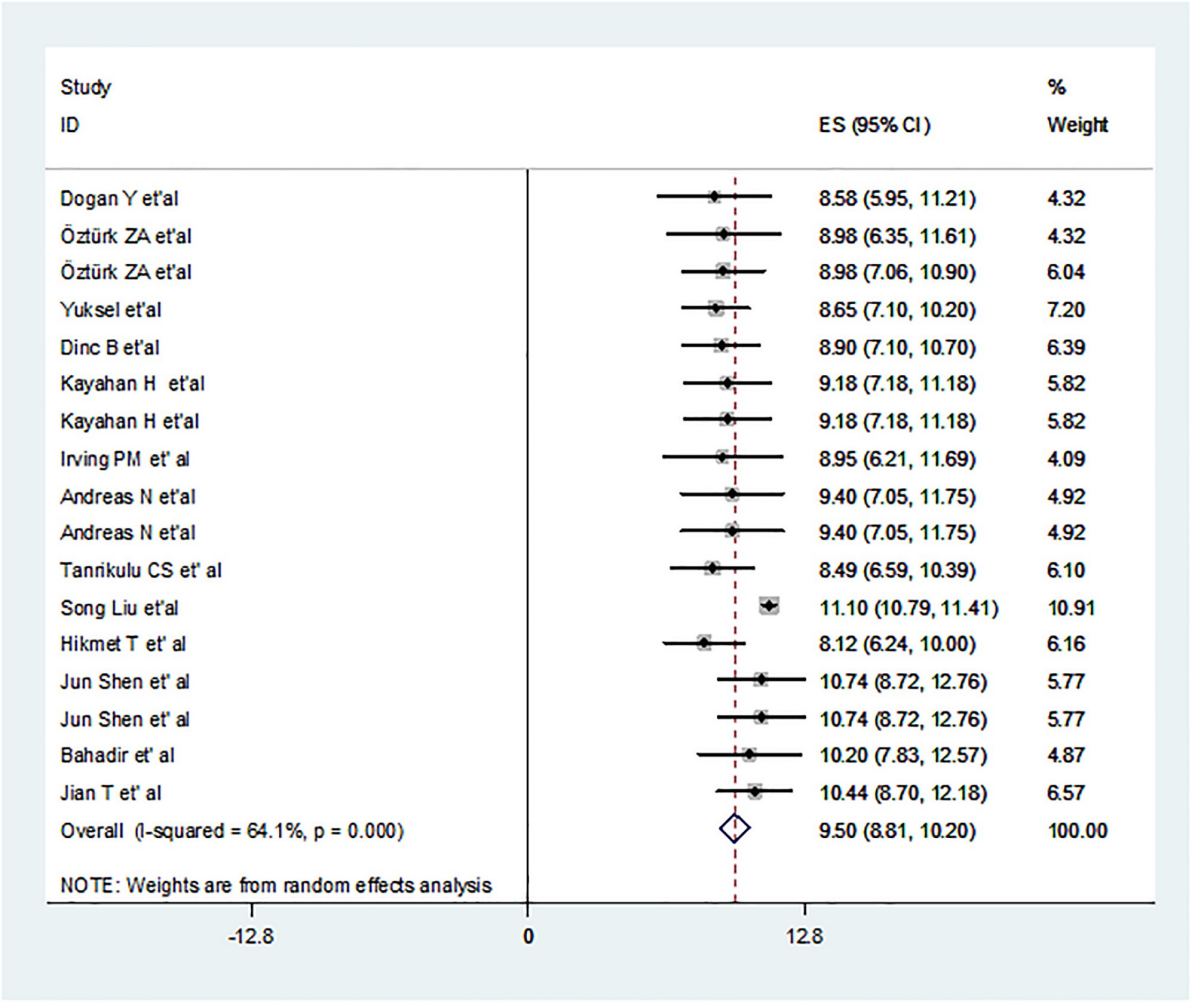
Forest plot of pooled estimated MPV in healthy controls. The size of the x-axis shows the estimate of pooled MPV of the studies. MPV is seen in the hard line (MPV = 0). In the pooled point calculation, the dotted line represents the MPV. The black dot in the middle of the gray box reflects the estimate pooled MPV of each studies point and the line shows the 95% CI of the estimates. The gray boxes represent each study weight that contributes to the estimation of the pooled MPV. I-squared illustrates the heterogeneity between the included studies.

In this study, heterogeneity was checked and a random effect model was applied. Based on the random effect model analysis, the overall pooled MPV were 9.29fl; 95% CI: 9.01–9.57 and 9.50fl; 95% CI: 8.81–10.20; p < 0.001 in IBD patients and control groups, respectively (Figs 3 and 4).

#### The pooled estimated mean difference in MPV

**Fig 5:**
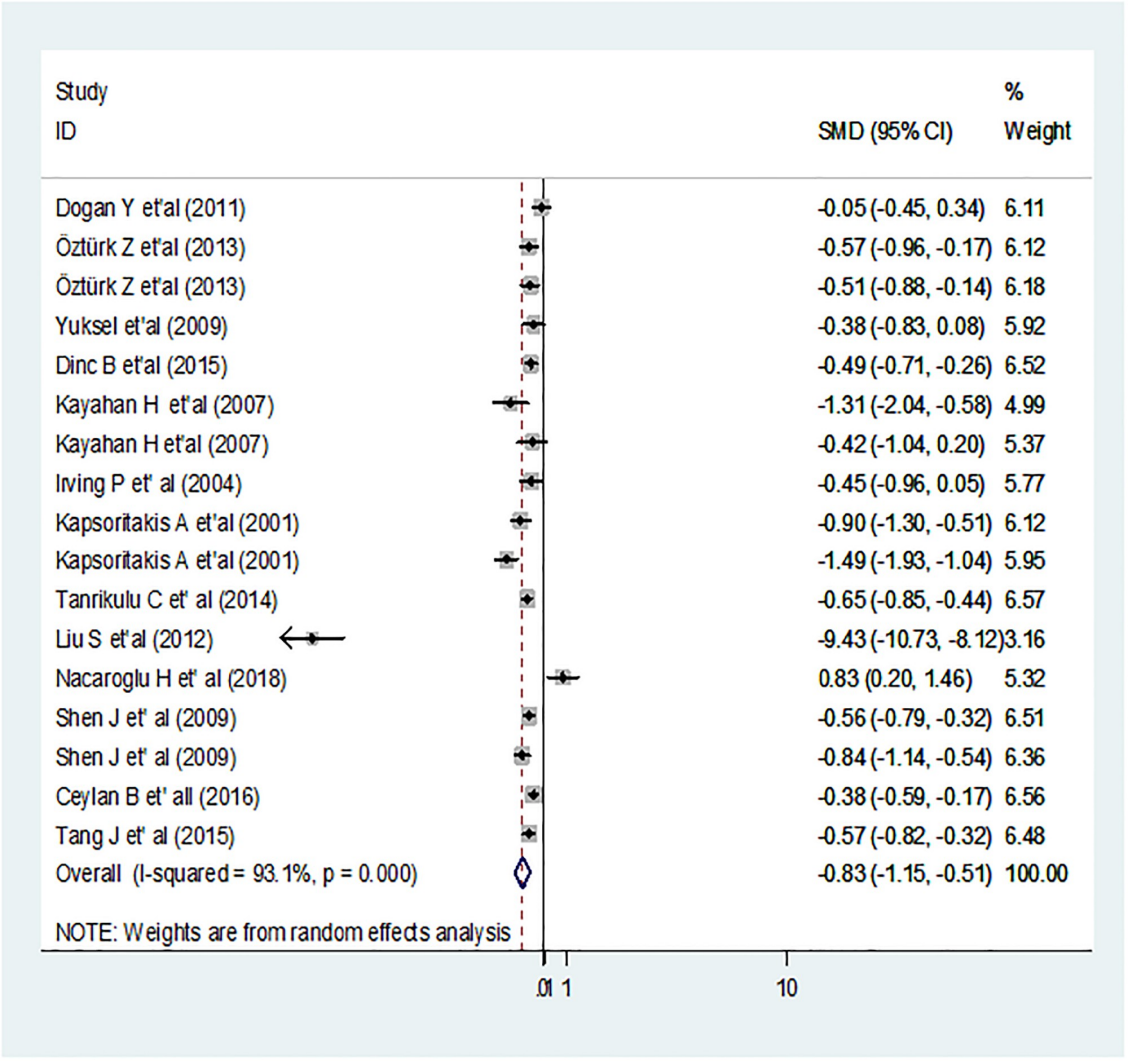
Forest plot of pooled estimated SMD using random effect model. The size of the x-axis shows the SMD estimate of the studies. Hard line indicates no difference (SMD = 0). In the pooled point calculation, the dotted line represents the mean difference. The black dot in the middle of the gray box reflects the SMD estimate of each sample’s point and the line shows the 95% CI of the estimates. The gray boxes represent each study weight that contributes to the estimation of the pooled mean difference. I-squared illustrates the heterogeneity between the included studies.

**Fig 6:**
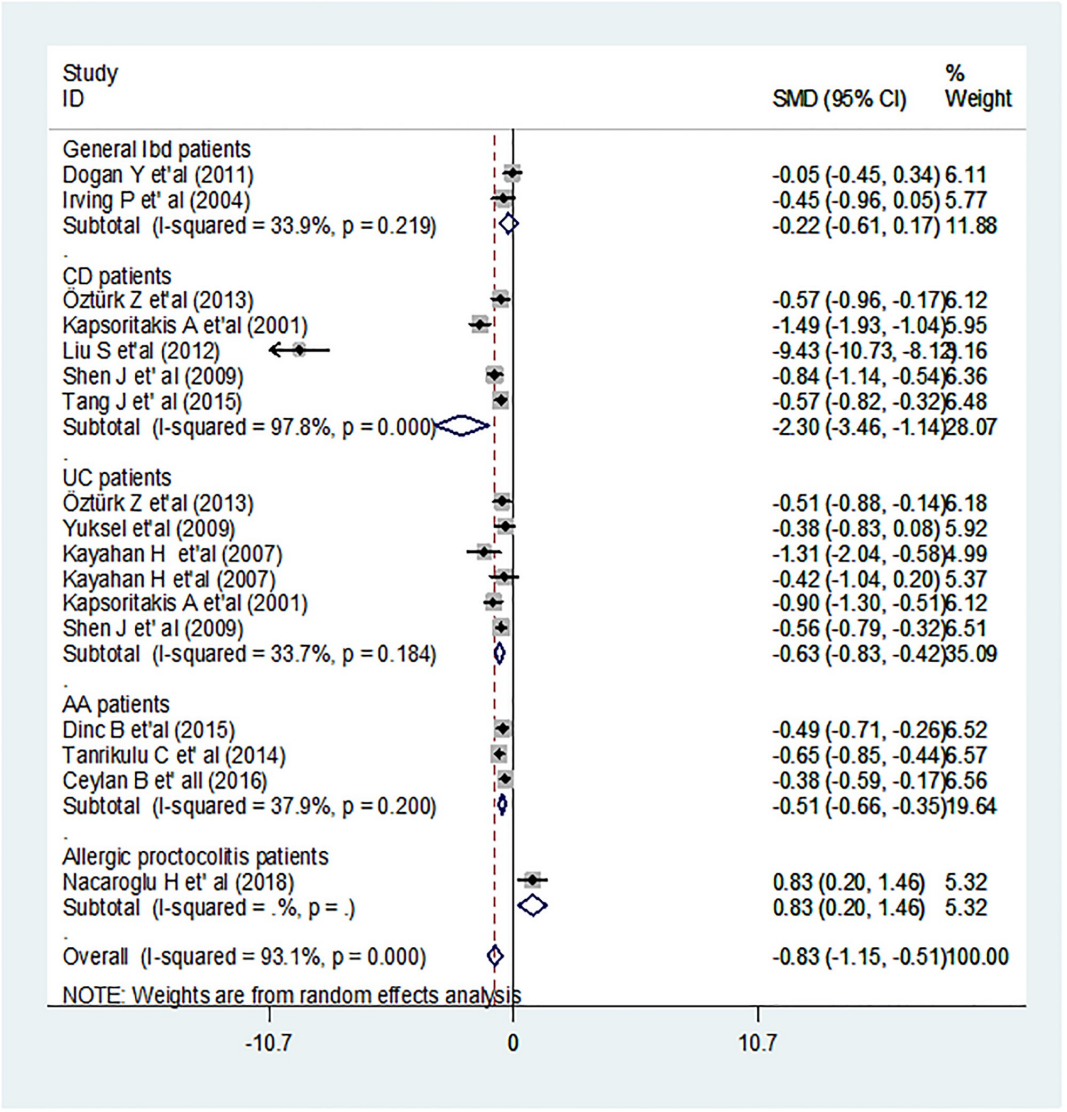
Forest plot of population based sub-group analysis. The x-axis scale displays the estimation of the SMD in MPV. The hard line shows no difference. The dotted line represents the mean difference in the pooled point estimate of each study. In the center of the gray box, the black dot reflects the SMD estimate of each article’s point estimate and the line shows the 95% CI of the estimates. I-squared indicates the heterogeneity across the included studies, p indicating for statistical significance of heterogeneity.

For each study, the mean difference in MPV between IBD and control was estimated. The pooled estimated SMD was -0.83fl; 95% CI: -1.15,-0.51; p < 0.001 (Fig 5). The finding was suggesting as the pooled average MPV in IBD patients was 0.83fl lower than the average pooled MPV of healthy controls. Since this pooling was extremely heterogeneous (I^2^ = 93.1%; p < 0.001) and so population based sub-group analysis was used to investigate potential sources of heterogeneity. The highest estimated mean difference in MPV and significant heterogeneity were observed among patients of CD; SMD = -2.30; 95% CI: -3.46, -1.14; I2 = 97.8%; p < 0.001 whereas the difference was lowest and insignificant heterogeneity in general IBD patients; SMD = -0.22; 95% CI: -0.61, -0.17; 33.9%; p = 0.98, UC; -0.63; 95% CI: -0.83, -0.42; I^2 =^ 33.7%; p = 0.18, AA; -51; 95% CI: -0.66, -0.35; p = 0.20 (Fig 6).

## Discussion

Platelet activation plays a critical role in thrombosis and inflammation in physiopathology. The RDW and MPV have been shown in several studies to be useful in the diagnosis of IBD. Both RDW and MPV are clinically significant hematologic markers that are routinely used in CBC. However, the efficacy of laboratory tests for IBD diagnosis has been poorly understudied. Meanwhile, MPV has long been used to measure platelet development in the bone marrow and has clinical relevance in some cases; MPV levels may be altered in hypertension, diabetes and IBD [13, 48–52]. But there is no well-established diagnostic value for MPV in IBD. Furthermore, it is very scant evidence of MPV in IBD in clinical practice. Therefore, this review would highlight accurate aggregated evidence of MPV in IBD.

In this study, 17 original articles conducted on different types of IBD namely active and inactive UC, general IBD, AA and CD were included. The pooled SMD in MPV = -0.83fl (95% CI: -1.15, -0.5; p-value < 0.001. It is fact that the pathophysiology IBD and MPV are biologically plausible. According to the finding, IBD patients had 0.83fl lower MPV as compared to healthy controls. It could be due to consumption or sequestration of platelets in the vascular segments associated with high grade of inflammation of the disease [53].

The finding was consistent with systematic review and meta-analysis conducted among patients of AA (weighted mean difference, -0.64; 95% CI, -0.74 to -0.54; *P* = 0.034 [54]. Furthermore, the pooled estimates of MPV in this study was in line with study in China, (MPV = 9.55 ± 0.17 in IBD and 11.1 ± 0.16 in healthy controls) [41]. The possible explanation would be similarity in intensity of systemic inflammatory response, consumption and sequestration of platelet during activation of the coagulation system [55]. On the other hand, the finding was in contradiction with a systematic review and meta-analysis conducted on coronary artery disease patients in Thailand [56] and neonatal sepsis; pooled mean difference in MPV were 0.84fl, 1.49fl, respectively [57]. The possible explanation might be disparity in disease pattern (low grade inflammatory response), population characteristic and micro-thrombi formation of platelet in the microvasculature related to bacteremia, cardiovascular risk factors and the other genetic factors, which result in increased MPV [58].

There was substantial heterogeneity in pooling SMD (I^2^ = 93.1%; p <0.001). Usually based on random effect model in population based subgroup analysis of mean difference in MPV, significant heterogeneity was observed in studies conducted among CD patients, (I^2^; 97.8%; p < 0.001). The possible explanation would be difference in study design, population, statistical methods, reference range, standard operating procedures and electronic cell counters. However, there was no substantial heterogeneity in studies conducted on general IBD, UC and AA. The reason may be due to similarity in cutoff value, study methodology, sample size, population characteristic and standard operating procedure.

### Implication in current clinical practice and future perspectives of MPV in IBD

While the pathogenesis of IBD still being understood, it has been proved that the release of inflammatory mediators via the activation of immune and coagulation pathways contributes to endothelial dysfunction, which is responsible for the disease’s clinical phenotype. Platelets were frequently consumed during thromboembolic events in association of the pathogenesis of IBD [19, 59]. The MPV correlated with function of platelet and is sensitive, specific and surrogate biomarker of many inflammatory disorders [60].

Platelets have both anti-inflammatory and anti-thrombotic properties. Systemic thromboembolism is more common in patients with IBD and multifocal micro vascular infarction has been suggested as a pathogenesis cause in CD. Meanwhile, increased platelet activation and aggregation are common features of IBD, which may lead to the risk of systemic thromboembolism and the pathogenesis of mucosal inflammation [19]. In active IBD, MPV is significantly decreased and it is negatively correlated with recognized IBD activity markers and platelet activation products [38].

The MPV and endoscopic activity index of IBD had a negative relationship (r: -0.358 p: 0.005) [39]. In contrary, it is inversely related with disease pattern and significantly correlated with endoscopic severity and histological activities of IBD [19]. Furthermore, thrombocyte count and MPV are considered as useful markers of IBD [20]. Several studies have indicated that platelets can play a role in CD pathogenesis and the MPV has been linked to CD and has been used as a possible inflammatory marker [61, 62]. In addition, it is fact that patients with CD had significant decreased MPV [40]. Furthermore, MPV is, non-invasive and available in CBC and used to discriminate cause of thrombocytopenia [63]. And also MPV was 76.6% reliable inflammatory marker in differentiating CD under Receiver operating characteristic (ROC) curve with a sensitivity and a specificity of 78.7% and 74.0%, respectively [42]. In addition, the overall accuracy of MPV in determining active UC was 71% with 67% sensitivity and 73% specificity [39].

Many literatures suggest that, MPV may provide valuable information on the course and prognosis in many pathological conditions, such as cardiovascular diseases, respiratory diseases, CD, rheumatoid arthritis, juvenile systemic lupus erythematous, diabetes mellitus, extreme infection, injuries, serious illness, trauma, systemic inflammatory response syndrome, thrombotic infections and the majority of neoplastic disease. The MPV levels were found to be lower in UC and increase in adult’s systemic lupus erythematous and a variety of neoplastic illnesses [60, 64–68]. Mean platelet volume is a routinely measured platelet size marker with established predictive value for a variety of cardiovascular disorders [56].

The longitudinal study estimated the promising efficacy of this marker as measured in the 24^th^-28^th^ gestational weekly interval suggested that the combined assessment of MPV in the first trimester was able to identify preeclampsia and intrauterine growth restriction with a sensitivity and specificity of 75% and 85.3%, respectively [69]. Furthermore, in this study MPV was significantly decreased in IBD.

#### Strength and limitation of the study

Articles were searched strategically and extensively through different searching engines. Moreover, the study was conducted in accordance to PRISMA guideline and protocols. However, there are potential limitations of the study. First, the articles used were published only in English and most of them were in Turkey, may cause geographical bias. The other limitation was platelet parameters underwent a number of changes in inflammatory disease. So, determining all platelet parameters would provide important diagnostic and prognostic information. However, included studies only reported MPV and other platelet parameters were not investigated in the study.

#### Conclusion and recommendation

The current meta-analysis demonstrated that MPV has decreased significantly in IBD with prominent mean difference, suggesting that MPV would represent the promising test in diagnosis and monitoring IBD. Therefore, MPV would be diagnostic and prognostic marker in clinical practice of IBD. Platelet indices would provide reliable information for assessing the severity of the disease and insight the possible pathophysiology of IBD. Therefore, before using single MPV as inflammatory marker, further cumulative evidence of all platelet indices are warranted. In addition, adequate clinical trials should be designed to establish the diagnostic and prognostic value of MPV as inflammatory marker in IBD.

## Supporting information

S1 Table The methodological quality of the included studies using JBI critical appraising tool. (DOCX)

S1 Checklist The PRISMA (Preferred reporting items for systematic and meta-analysis) checklists. (DOCX)

S1 Data (XLSX)
