# Supplementary material for: A mean platelet volume in inflammatory bowel disease: A systematic review and meta-analysis
Source: PLoS One. 2022 Aug 30;17(8):e0273417. doi: 10.1371/journal.pone.0273417 (PMC9426900; doi:10.1371/journal.pone.0273417)
Supplement: S1 Table — (DOCX) [file pone.0273417.s001.docx]

Supplementary table 1: The methodological quality of the included studies using JBI critical appraising tool

| **Study ID** | Representation | **Sampling** | **Comparability** | **Eligibility protocols** | **Data collection** | **Description of study subject** | **Reliable and valid outcome measurement** | **Bias minimization** | **Overall quality summary** |
| --- | --- | --- | --- | --- | --- | --- | --- | --- | --- |
| Irving P et' al ([35](file:///C:\Users\User\Desktop\Track%20changed%20%20manuscript.docx#_ENREF_35)) | 1 | 1 | NA | 1 | 1 | 1 | 1 | UC | High |
| Öztürk Z et'al ([36](file:///C:\Users\User\Desktop\Track%20changed%20%20manuscript.docx#_ENREF_36)) | 1 | 1 | 1 | 1 | 0 | 1 | 1 | 1 | High |
| Öztürk Z et'al ([36](file:///C:\Users\User\Desktop\Track%20changed%20%20manuscript.docx#_ENREF_36)) | 1 | 1 | 1 | 1 | 0 | 1 | 1 | 1 | High |
| Kayahan H et'al ([37](file:///C:\Users\User\Desktop\Track%20changed%20%20manuscript.docx#_ENREF_37)) | 1 | 1 | 1 | 1 | 1 | 1 | 1 | 1 | High |
| Kayahan H et'al ([37](file:///C:\Users\User\Desktop\Track%20changed%20%20manuscript.docx#_ENREF_37)) | 1 | 1 | 1 | 1 | 1 | 1 | 1 | 1 | High |
| KapsoritakisA et'al ([38](file:///C:\Users\User\Desktop\Track%20changed%20%20manuscript.docx#_ENREF_38)) | 1 | 1 | 1 | 1 | 1 | 1 | 1 | 1 | High |
| Kapsoritakis A et'al ([38](file:///C:\Users\User\Desktop\Track%20changed%20%20manuscript.docx#_ENREF_38)) | 1 | 1 | 1 | 1 | 1 | 1 | 1 | 1 | High |
| Yuksel et'al ([39](file:///C:\Users\User\Desktop\Track%20changed%20%20manuscript.docx#_ENREF_39)) | 1 | 1 | NA | 1 | 1 | 1 | 1 | 0 | High |
| Shen J et' al ([40](file:///C:\Users\User\Desktop\Track%20changed%20%20manuscript.docx#_ENREF_40)) | 0 | 1 | 1 | 1 | 1 | 0 | 1 | 0 | Good |
| Shen J et' al ([40](file:///C:\Users\User\Desktop\Track%20changed%20%20manuscript.docx#_ENREF_40)) | 1 | 1 | 1 | 1 | 1 | 1 | 1 | 1 | High |
| Liu S et'al ([41](file:///C:\Users\User\Desktop\Track%20changed%20%20manuscript.docx#_ENREF_41)) | 0 | 1 | NA | 1 | 1 | 1 | 1 | UC | Good |
| [Tang J et' al](https://pubmed.ncbi.nlm.nih.gov/?term=Tang+J&cauthor_id=25565427)([42](file:///C:\Users\User\Desktop\Track%20changed%20%20manuscript.docx#_ENREF_42)) | 1 | 1 | 1 | 1 | 1 | 1 | 1 | 1 | High |
| Dogan Y et'al ([43](file:///C:\Users\User\Desktop\Track%20changed%20%20manuscript.docx#_ENREF_43)) | 1 | 1 | 1 | 1 | 1 | 1 | 1 | 1 | High |
| Nacaroğlu H et' al ([44](file:///C:\Users\User\Desktop\Track%20changed%20%20manuscript.docx#_ENREF_44)) | 1 | 1 | NA | 1 | 1 | 0 | 1 | 0 | Good |

Supplementary table 1: (Continued)

| Tanrikulu C et' al ([45](file:///C:\Users\User\Desktop\Track%20changed%20%20manuscript.docx#_ENREF_45)) | 0 | 1 | NA | 1 | 1 | 1 | 1 | 0 | Good |
| --- | --- | --- | --- | --- | --- | --- | --- | --- | --- |
| Ceylan B et' all ([46](file:///C:\Users\User\Desktop\Track%20changed%20%20manuscript.docx#_ENREF_46)) | 1 | 1 | 1 | 1 | 1 | 1 | 1 | UC | High |
| Dinc B et'al ([47](file:///C:\Users\User\Desktop\Track%20changed%20%20manuscript.docx#_ENREF_47)) | 1 | UC | 1 | 1 | 1 | 1 | 1 | 0 | Good |

**NB.** Overall quality summary; combined methodological quality for all included research designs; Good quality (50-75%) score; High quality (50-7 %) score, (1) for done, (0) Not done; **UC**: unclear; **NA**: not applicable.
