# Supplementary material for: A mean platelet volume in inflammatory bowel disease: A systematic review and meta-analysis
Source: PLoS One. 2022 Aug 30;17(8):e0273417. doi: 10.1371/journal.pone.0273417 (PMC9426900; doi:10.1371/journal.pone.0273417)
Supplement: S1 Checklist — (DOCX) [file pone.0273417.s002.docx]

The PRISMA (Preferred Reporting Items for Systematic and Meta-Analysis)  checklists

| **Section and topic** | **Check list No** | **Checklist items** | **Page numbers (s)** |
| --- | --- | --- | --- |
| **Title** | | |  |
| Identification |  | Identify the report as a systematic review, meta- analysis, or both. | 1 |
| Update |  | If the protocol is for update of previous systematic review identify as such | N/A |
| **Registration** |  | If registered, provide the name of registry ( e.g. PRESPERO) and registration number in the abstract | 2 |
| **Introduction** | | | |
| Rationale |  | Describe the rationale for the review in the context of what is already known | 4 |
| Objectives |  | Provide an explicit statement of the question(s) the review will address with reference to participants, interventions, comparators, and outcomes (PICO) | 4 |
| **METHODS** | | | |
| Eligibility criteria |  | Specify the study characteristics (such as PICO, study design, setting, time frame) and report characteristics (such as years considered, language, publication status) to be used as criteria for eligibility for the review | 6 |
| Information sources |  | Describe all intended information sources (such as electronic databases, contact with study authors, trial registers or other grey literature sources) with planned dates of coverage | 5 |
| Search strategy |  | Present draft of search strategy to be used for at least one electronic database, including planned limits, such that it could be repeated | Supplemental file 2 |
| Data management |  | Describe the mechanism(s) that will be used to manage records and data throughout the review | 7 |
| Selection process |  | State the process that will be used for selecting studies (such as two independent reviewers) through each phase of the review (that is, screening, eligibility and inclusion in meta-analysis) | 7 |
| Data collection process |  | Describe planned method of extracting data from reports (such as piloting forms, done independently, in duplicate), any processes for obtaining and confirming data from investigators | 8 |
| Data items |  | List and define all variables for which data will be sought (such as PICO items, funding sources), any pre-planned data assumptions and simplifications | 6,9 |
| Outcomes and prioritization |  | List and define all outcomes for which data will be sought, including prioritization of main and additional outcomes, with rationale | 6 |
| Risk of bias in individual studies |  | Describe anticipated methods for assessing risk of bias of individual studies, including whether this will be done at the outcome or study level, or both; state how this information will be used in data synthesis | 7 |
| **Data** | | | |
|  |  | Describe criteria under which study data will be quantitatively synthesized | N/A |
| **Synthesis** |  | If data are appropriate for quantitative synthesis, describe planned summary measures, methods of handling data and methods of combining data from studies, including any planned exploration of consistency (such as I^2^, Kendall’s τ) | N/A |
|  |  | Describe any proposed additional analyses (such as sensitivity or subgroup analyses, meta-regression) | 9 |
|  |  | If quantitative synthesis is not appropriate, describe the type of summary planned | 9 |
|  |  | Give results of additional analyses, if done (e.g., sensitivity or subgroup analyses, meta-regression | N/A |
| **Discussion** | | | |
| Summary of evidence |  | Summarize the main findings including the strength of evidence for each main outcome; consider their relevance to key groups (e.g., healthcare providers, users, and policy makers). | N/A |
| Limitations |  | Discuss limitations at study and outcome level (e.g., risk of bias), and at review-level (e.g., incomplete retrieval of identified research, reporting bias). | N/A |
| Conclusion |  | Provide a general interpretation of the results in the context of other evidence, and implications for future research. | N/A |
| **Funding** | | | |
| Fund |  | Describe sources of funding for the systematic review and other support (e.g., supply of data); role of funders for the systematic review. | 10 |
